# Scintillations of Similia

**Published:** 1867-07

**Authors:** 


### *Scintillations of Similia.*

A prominent business man of this city, whose name will be given if the infinitesimals so desire, assured us, in private conversation a few days since, that he was formerly very subject to *bilious* attacks, for which the "Allopaths" always gave him blue pill or calomel. Now he was under homœopathic treatment, (Dr. M—r,) and all he was directed to do was to take a teaspoonful of concentrated essence of Jamaica ginger and three tablespoonfuls of Bourbon whiskey at a dose. This cured him *every time.*

His little child had convulsions, and the same homœopath gave it full doses of calomel and rhubarb, with successful result.

Such cases are numberless, within our own observation. Meanwhile we are told—"Homœopathy is spreading and growing! *Vive la bagatelle!*

